# Human behavior alignment to improve the robustness of deep neural networks

**DOI:** 10.3389/frai.2026.1709840

**Published:** 2026-08-19

**Authors:** Bharath Anand, Sarada Krithivasan

**Affiliations:** 1 Elmore Family School of Electrical and Computer Engineering, Purdue University, West Lafayette, IN, United States; 2 IBM T. J. Watson Research Center, Yorktown Heights, NY, United States

**Keywords:** deep neural networks, human behavior alignment, label smoothing, robustness, training

## Abstract

Deep neural networks (DNNs) are known to produce erroneous results under real-world noisy inputs, presenting a major bottleneck to their use in applications where lives, safety, or significant resources are at stake. It has been commonly observed that humans are highly resilient to the noisy inputs that are challenging for DNNs. However, very few efforts have translated this observation into techniques to improve DNN robustness. We hypothesize that statistically aligning DNNs to human behavior during training could improve robustness. Based on this insight, we propose BrainTrain, a framework to create more robust DNNs through human behavior alignment and demonstrate its utility in the context of object recognition. BrainTrain captures human behavior in the form of a confusion matrix constructed from human responses to object recognition challenges and uses a composite loss function to co-optimize accuracy and human behavior alignment during stochastic gradient descent (SGD) based training. We also propose Similarity Driven Label Smoothing (SDLS), a regularization method that scales BrainTrain to applications where it is difficult or expensive to collect human behavioral data. DNNs trained with BrainTrain showed up to 26% higher accuracy under a wide range of noisy inputs and 2.1 times lower calibration error with negligible increase in training time. We also demonstrate that SDLS leads to improvements in noise robustness in ResNets trained on the ImageNet-1 K image classification dataset. We show that BrainTrain is complementary to conventional techniques like noise-added training and can provide further improvements over and above these techniques.

## 1 Introduction

Recent advances in Artificial Intelligence (AI) have been driven by deep neural networks (DNNs), leading to remarkable improvements in many tasks including computer vision and natural language processing (Hassabis et al., 2017; Macpherson et al., 2021; Roser, 2022). With improvements in DNN capabilities, there is increasing interest in using them in critical applications such as healthcare, autonomous vehicles, law enforcement and defense. Indeed, DNNs are already deployed in autonomous vehicles and unmanned aerial vehicles (UAVs), diagnostic radiology and criminal justice. A major challenge to the use of DNNs in such applications is their susceptibility to failure in the presence of input noise and variations, where errors can put lives, safety, or significant resources at risk. For example, previous work has shown that medical imaging DNNs considerably degrade in accuracy in the presence of input noise (Tian et al., 2021; Phillips et al., 2020). It has also been demonstrated that DNNs used for autonomous driving may make incorrect, catastrophic decisions when presented with noisy inputs (Tian et al., 2018; Eykholt et al., 2018). A comprehensive characterization of DNNs under input noise indicates that accuracy degrades considerably under a wide range of synthetic noise that reflect noise observed in real-world settings (Hendrycks and Dietterich, 2019). In summary, improving the robustness of DNNs is a critical challenge that needs to be addressed to enable their broader adoption. While robustness can be addressed with respect to naturally occurring or deliberately crafted adversarial noise, our focus is primarily on naturally occurring noise, since it occurs pervasively in real-world conditions (Zheng et al., 2016).

Our work is motivated by the observation that humans are often not susceptible to noisy inputs that make DNNs fail. While human robustness to noisy inputs is well-known, bridging the DNN-human robustness gap by explicitly learning from human behavior remains an open challenge. To that end, we propose human behavior alignment as a method to impose a human-informed inductive bias during DNN training. In human behavior alignment, the DNN training process is modified to explicitly optimize for similarity to human behavior in addition to task accuracy. Specifically, we propose to use human responses to time-constrained object recognition challenges to capture human behavior. These responses are represented as a class confusion matrix, which is used derive soft labels that are added to the loss function during DNN training. We implemented this approach, called BrainTrain, within PyTorch and used it to train optimized image classification models based on the widely used ResNet family of DNNs. We evaluated the models on inputs with various types and levels of noise. Models trained with BrainTrain showed considerable improvement in robustness to noise and calibration (the ability to provide a reliable probability of correctness for the model’s predictions).

We also created ORCA (Object Recognition Challenge App), a cross-platform mobile application (Android and iOS) to enable further human behavior data collection. The ORCA app presents users with a query image followed by two choices, one of which contains the same object as the query. The app only collects challenge responses without storing any private user data.

Finally, to scale our approach to applications where human behavioral data may be challenging to obtain, we propose Similarity Driven Label Smoothing (SDLS), a variant of teacher-student learning where the confusion matrix is created using a large teacher model and used in the training of a robust student model. We evaluate this approach on ResNet models trained for the ImageNet-1 K dataset and demonstrate improvements in baseline models trained with conventional SGD as well as noise-added training.

### 1.1 Related work

Previous research has extensively explored the adverse effect of input noise on DNN performance in various application domains as well as several approaches to mitigate it. For example, Tian et al. (2021) showed that bias fields that can occur because of improper image acquisition in medical imaging can lead to incorrect classifications by DNNs, while Phillips et al. (2020) explores the impact of various real-life noise sources on chest X-ray classification DNNs. Tian et al. (2018) evaluates DNNs used in autonomous driving and demonstrates that there is a significant chance of catastrophic decisions such as steering into oncoming traffic under noisy inputs. Zheng et al. (2016) shows that image processing artifacts such as compression cause significant instability in DNN model outputs, i.e., different outputs for visually similar or indistinguishable images. Hendrycks and Dietterich (2019) studies the robustness of image classifiers under a broad range of naturally occurring input noise.

Previous approaches to improving DNN robustness include noise-added training (augmenting the training dataset with inputs that contain noise) (Zheng et al., 2016), other data augmentation techniques such as input mixing (Yun et al., 2019; Gowal et al., 2020), and pre-processing inputs during inference to detect and/or remove noise (Maas et al., 2012). While each of these approaches has shown promise, they also suffer from various drawbacks such as an increase in training or inference time, or a negative impact on clean accuracy under noise-free inputs.

Label smoothing (Szegedy et al., 2016; Müller et al., 2019) is a regularization technique that was proposed to improve generalization and robustness, specifically to noisy labels (mislabeled training data). It has also been shown to reduce overconfidence in model predictions (Müller et al., 2019). In label smoothing, the hard target labels are replaced with soft targets that are computed as a weighted average of the hard targets and a uniform distribution over all labels (Müller et al., 2019). Recent variants of label smoothing have explored assigning soft targets based on class similarity with the correct class, demonstrating further improvements in calibration (Liu et al., 2021). We note that in contrast to prior work on label smoothing, our work focuses on robustness to input noise. Additionally, our results demonstrate that BrainTrain is more effective in improving input noise robustness than label smoothing.

Recent efforts have explored the design of more “brain-like” DNNs, for example by adding a brain-like V1 layer at the inputs or by encouraging internal representations to be more similar to neural recordings from primates (Dapello et al., 2020). In contrast to these efforts, our approach does not modify the neural network architecture, nor does it require the collection of neural recordings, which in turn requires the implantation of electrodes, greatly limiting applicability in humans. Another related line of work aims to create models of human perception from DNNs. To that end, Lee (2022) proposed adding a linear layer that projects their final representation onto a much smaller number of dimensions such that predictivity of human behavior was maximized. Our work aims to improve DNN robustness as opposed to their ability to predict human behavior.

In contrast with previous approaches that invariably involve significant tradeoffs in training time or clean accuracy, BrainTrain does not suffer from these drawbacks. Further, we demonstrate that BrainTrain can be combined with previous methods such as noise-added training to achieve additional improvements in noise robustness.

## 2 Methods

**Figure 1:**
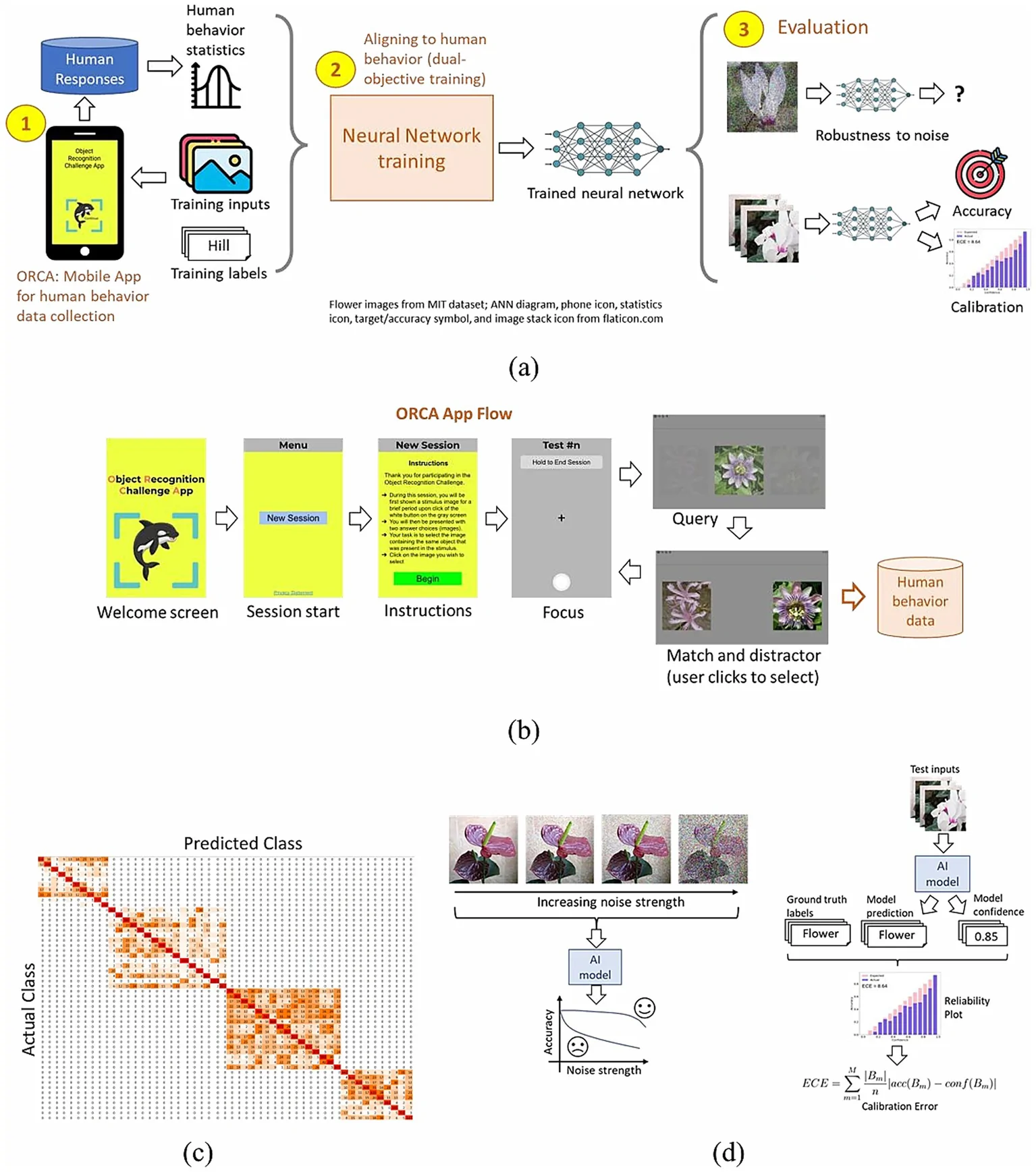
**(a)** Overview of the BrainTrain framework; **(b)** Overview of Object Recognition Challenge App (ORCA), a cross-platform mobile application that enables human behavior data collection by presenting users with a series of image similarity judgment tasks. The user is presented with a query image followed by two choices—a match and a distractor—and asked to pick the one that best resembles the query; **(c)** confusion matrix computed from 48,689 human responses to image similarity judgment tasks. The rows represent the true class of an image, while the columns represent the class that the human associated the image with. Entries are color-coded with darker shades representing higher probabilities; **(d)** Evaluation framework for BrainTrain. We evaluate accuracy under varying types and levels of noise as well as calibration error, a metric that quantifies how well the DNN’s confidence in its outputs matches with the actual probability of correctness. Images adapted with permission from the Oxford Flowers 102 dataset, https://www.tensorflow.org/datasets/catalog/oxford_flowers102, Nilsback and Zisserman (2008), licensed under CC-BY 4.0.

BrainTrain is a framework to create robust DNNs by aligning them with human behavior. BrainTrain consists of three steps, as outlined in Figure 1a.

- The first step involves collecting human behavioral data. This requires presenting the task that the DNN is supposed to solve to humans and collecting their responses. In the context of object recognition, which is the focus of our work, we adopt an approach that is common in cognitive science, which is to collect human similarity judgements (Lee, 2022).
- The second step involves using human behavioral data to train DNNs such that their predictions are statistically aligned to human behavior. Towards this end, we propose to quantify human behavior alignment as the L2 distance between the class probabilities predicted by the DNN and the class probabilities computed from the human behavioral data. We modify the loss function used in DNN training to be a composite loss that combines a task accuracy term (e.g., cross entropy) and a human behavior alignment term.
- The third step involves evaluating the DNNs produced by the BrainTrain framework to quantify their benefits over baseline DNNs obtained from standard training. We present the models with inputs containing various types and levels of noise and measure metrics such as accuracy under noisy inputs and calibration error.

Each of these steps is described in detail in the following subsections.

### 2.1 Human behavioral data

Creating human behavior aligned DNNs requires that we collect human behavioral data and represent it using suitable statistical metrics that can be utilized during training. In this context, it should be noted that labeled datasets used for supervised learning already contain human input in the form of the target labels. We use the term human behavior to refer to data that goes beyond just the correct class labels. We present human subjects with image similarity judgment tasks created from labeled data, i.e., images for which correct class labels are known. The approach of using similarity judgment tasks to capture human behavior has been widely used in cognitive science (Lee, 2022). The approach suggested in Lee (2022) involves presenting the subject with a forced-choice similarity judgment task involving triplets of stimuli (as opposed to the classical approach of pairs of stimuli) under stringent timing constraints. Users are presented with a query image for a very brief period and showed two answer choice images. The user’s task is to select the image with the same object as the initial image. This methodology sidesteps issues involving pairwise ratings such as the variation in how humans scale their ratings (Lee, 2022). Each human response is in the form of a 4-tuple {*Q*, *M*, *D*, *R*}, where *Q* is the class label corresponding to the query image, *M* is class label corresponding to the match image, *D* is the class label corresponding to the distractor image, and *R* is the user response that can be equal to 1 or 0 depending on whether the user picked the match image (correct response) or distractor image (incorrect response).

From the human responses, we construct a confusion matrix that captures how often objects of each class were classified as belonging to the correct class as well as each other class. The following pseudocode describes the procedure for constructing the confusion matrix.

Algorithm 1: **Construct human behavior confusion matrix**

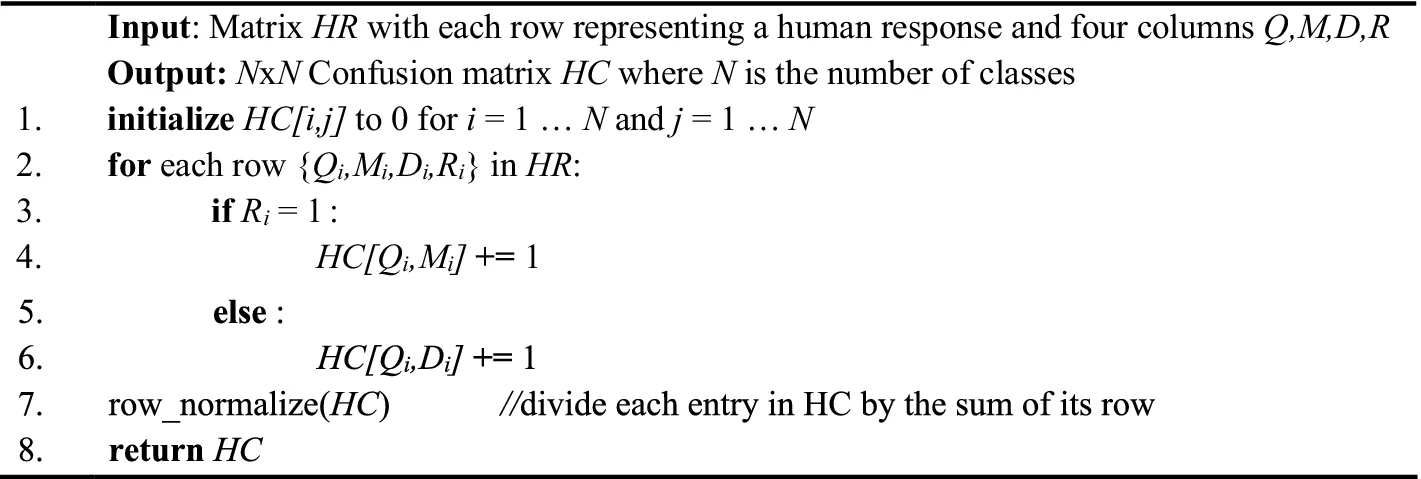

Figure 1c shows a confusion matrix computed from a dataset of 48,689 human responses to object recognition challenges from the authors of (Lee, 2022) that were collected through Amazon Mechanical Turk. The matrix has 52 rows and 52 columns, corresponding to the 52 classes in the dataset. We note that the confusion matrix has a block-diagonal structure, with four groups of classes that have non-zero entries and considerable variations in the entries within each group (represented by the varying shade of orange). We use the normalized entries of this confusion matrix as soft labels during DNN training.

We performed a bootstrap variance analysis (*N* = 1,000 resamples) to estimate the stability of the confusion matrix. The analysis revealed that the human confusion patterns are highly stable. The average standard deviation across diagonal elements (correct classifications) was 1.07%. The average standard deviation across off-diagonal elements (inter-class confusions) was 0.08%. The maximum standard deviation observed for any single off-diagonal element was 0.76%. These results confirm that the information derived from the human confusion matrix is statistically robust.

To facilitate further human behavioral data collection, we developed Object Recognition Challenge App (ORCA), an open-source cross-platform mobile app described in Figure 1b. The app presents users with a query image followed by a pair of images called the match and distractor. As the names suggest, the match image contains the same class of object as the query image, while the distractor contains a different class of object. The user is prompted to pick the image that matches the query. The app records challenge responses without storing any private user data. The app has been open-sourced with code available at: https://snack.expo.dev/@bharath.anand/object-recognition-challenge-app---orca?platform=android.

### 2.2 Training DNNs for human behavior alignment

Conventional DNN training minimizes a loss function that represents the accuracy of the DNN at performing the task at hand. For example, in the case of classification DNNs, it is common to use the cross-entropy loss function, which is given by Equation (1).


$$L_{\mathrm{CE}}(y,\hat{y})=-\sum_{k=1}^{N}\hat{y}[k]\log y[k]$$ (1)

In the above equation, *y* is a vector representing the class probabilities output by the DNN (the length of this vector is equal to the number of classes, N). *ŷ* is a one-hot vector that represents the ground truth or correct label (exactly one of the entries, corresponding to the correct class, is 1, while the other entries are 0).

BrainTrain uses a composite loss function that combines an accuracy-driven loss term with a novel term that minimizes deviation from human behavior. Specifically, we use the mean-squared error between the class probabilities computed by the DNN for a given input and the corresponding class statistics from the confusion matrix:


$$L_{\mathrm{HB}}(y)=\frac{1}{N}\sum_{k=1}^{N}{\left(y[k]-y_{\mathrm{HB}}[k]\right)}^{2}$$ (2)

In Equation (2), y is once again a vector representing the class probabilities output by the DNN, while *y_HB_* is a vector of target class probabilities derived from the human behavioral data. Specifically, *y_HB_* is the row of the confusion matrix corresponding to the target class of the current input.

The two loss terms are combined using the following equation to compute the overall loss function that is minimized during DNN training:


$$\text{Loss}=(1-\alpha )\times L_{\mathrm{CE}}+\alpha \times L_{\mathrm{HB}}$$ (3)

In Equation (3), *α* is a hyper-parameter that controls the relative strength of the two loss terms. A higher value of α corresponds to a higher emphasis on human behavior alignment, while an α of 0 defaults to the traditional cross-entropy loss function that optimizes for task accuracy.

### 2.3 Similarity driven label smoothing (SDLS)

**Figure 2:**
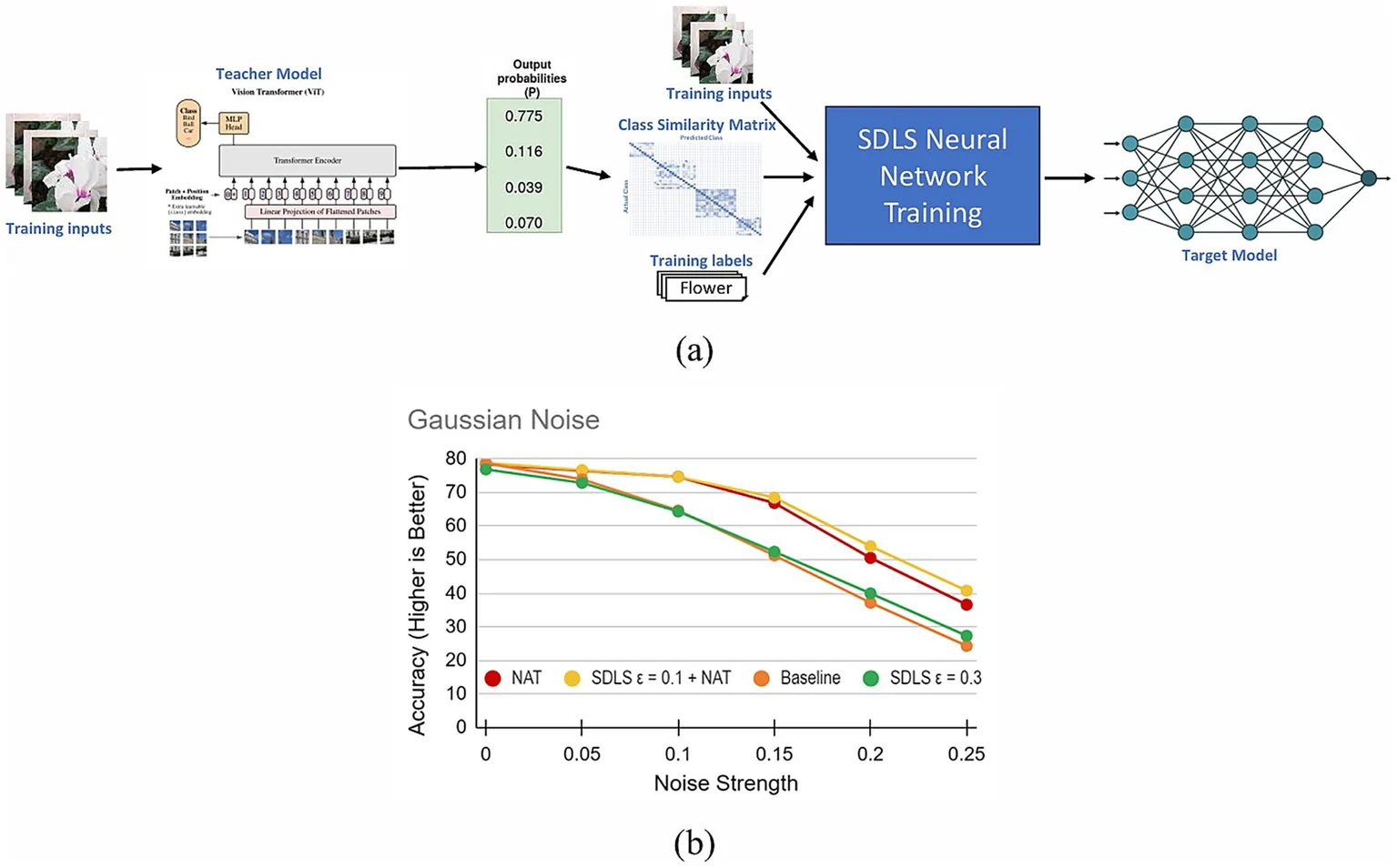
**(a)** Similarity-driven label smoothing (SDLS), in which a teacher model is used to generate a class similarity matrix that is used for training a target model (images adapted with permission from the Oxford Flowers 102 dataset, https://www.tensorflow.org/datasets/catalog/oxford_flowers102, Nilsback and Zisserman [2008], licensed under CC-BY 4.0); **(b)** accuracy under noise for ResNet-50 on the ImageNet-1 K dataset when trained with conventional SGD training (orange curve), SDLS (green curve), noise-added training (red curve), and SDLS with noise-added training (yellow curve).

The collection of human behavioral data can be time consuming for some applications. To address this challenge, we view BrainTrain through the lens of label smoothing (Szegedy et al., 2016; Müller et al., 2019), a regularization technique in which hard labels, represented by a one-hot vector of class probabilities for each input, are “smoothed” by assigning a small, non-zero probability to the incorrect classes. We observe that BrainTrain can be considered a form of label smoothing where the smoothing level for each class is determined by the confusion matrix of human responses. That is, the classes that are more frequently confused with the true class by humans are weighted higher in the smoothing. Inspired by this observation, we propose Similarity Driven Label Smoothing (SDLS), in which a large teacher model (such as a pretrained vision transformer in our experiments) is used to generate a confusion matrix, and the generated confusion matrix is used instead of human behavioral data in the BrainTrain training step. This process is illustrated in Figure 2a. Note that although we use the term “teacher” to refer to the model used to generate the confusion matrix, SDLS differs from teacher-student learning where the teacher’s outputs (and in some cases, internal representations) for each sample are used to train the student model. In contrast, we are only using the confusion matrix aggregated over all samples in the training dataset.

### 2.4 Evaluation framework

To determine whether BrainTrain achieves the desired goals, we developed a framework to evaluate the resulting DNNs and compare them to various baselines (Figure 1d). We perform two sets of experiments – one without and the other with data set augmentation. For the first experiment without dataset augmentation, we compare BrainTrain to the following baselines: Standard SGD, label smoothing (Szegedy et al., 2016), and class-based label smoothing (Liu et al., 2021). In these experiments, no noise is added to the training data. For the experiments with data set augmentation, we demonstrate that BrainTrain can be combined with noise-added training (BrainTrain+NAT) and compare this with two baselines—input mixing with CutMix (Yun et al., 2019), AutoAugment (Cubuk et al., 2019), and noise-added training (NAT) (Zheng et al., 2016) alone. For NAT and BrainTrain+NAT, only one type of noise (salt & pepper) was added during training in order to evaluate whether the models are robust to unseen noise types.

#### 2.4.1 Statistical evaluation methods

We tested all DNNs with inputs that contained different types and levels of noise. Following previous efforts (Tian et al., 2018), we considered the following five types of noise: (i) Salt & Pepper (S&P) noise, which represents the effect of bit-errors, (ii) Speckle noise, which is the main type of noise found in medical ultrasonic images, (iii) Gaussian noise, which is typical of images captured in low lighting conditions, (iv) Gaussian Blur, which mimics defocused images, and (v) Compression noise, which reflects the impact of image compression. We note that these noise types are reflective of input perturbations seen in real-world settings (Katta et al., 2025; Li et al., 2025). For each noise type, we varied numerical parameters that represent the noise strength. Robustness results were evaluated under 10 different random seeds, with standard deviations presented as error bands.

We report two metrics in our experiments – accuracy of the models under noisy inputs, and the expected calibration error (ECE), which quantifies how well the DNN’s confidence estimates match the actual probability of correctness (i.e., whether the model is over-confident or under-confident). ECE was calculated using 10 bins, and no models incorporated temperature scaling.

## 3 Results

BrainTrain was implemented in Python using open-source libraries including PyTorch (Paszke et al., 2019), scikit-image (van der Walt et al., 2014) and PIL (Umesh, 2012). For our experiments, we used a workstation with an Intel Xeon Silver 4114 CPU and an NVIDIA GTX 1080Ti GPU for all training, and a Lenovo Yoga 9i laptop with an Intel Core i7 CPU and 16GB RAM for evaluating the resulting DNNs. We used a dataset of 48,689 human responses to object recognition challenges from (Lee, 2022) and the corresponding images (256 × 256 resolution, belonging to 52 classes) were split in the ratio 65%:15%:20% for train/validation/test. We use two versions of ResNet, viz. ResNet-18 and ResNet-50 (He et al., 2016), which are pre-trained on ImageNet-1 K and fine-tuned for our experiments. Fine-tuning was conducted using SGD, with a learning-rate of 0.01 that decays by a factor of 10 every 30 epochs, momentum of 0.9, and weight decay of 1E-4 and batch-size of 64. For BrainTrain, we swept the value of the hyperparameter *α* that represents the strength of the human behavior alignment term in the loss function and found that 0.2 consistently gave us the best results. For standard label smoothing, we obtained best results when using the soft target 0.85 for the correct class and 0.15 evenly spread among all incorrect classes. For class-based label smoothing, the soft target was 0.85 for the correct class, 0.1 spread evenly among all classes in the same super-class as the correct class and 0.05 evenly spread across all other classes. The baseline model, both variants of label smoothing, and BrainTrain were fine-tuned for 6 epochs. The DNNs trained with CutMix, noise-added training, and BrainTrain combined with noise-added training were fine-tuned for 40 epochs due to the increase in dataset size.

**Figure 3:**
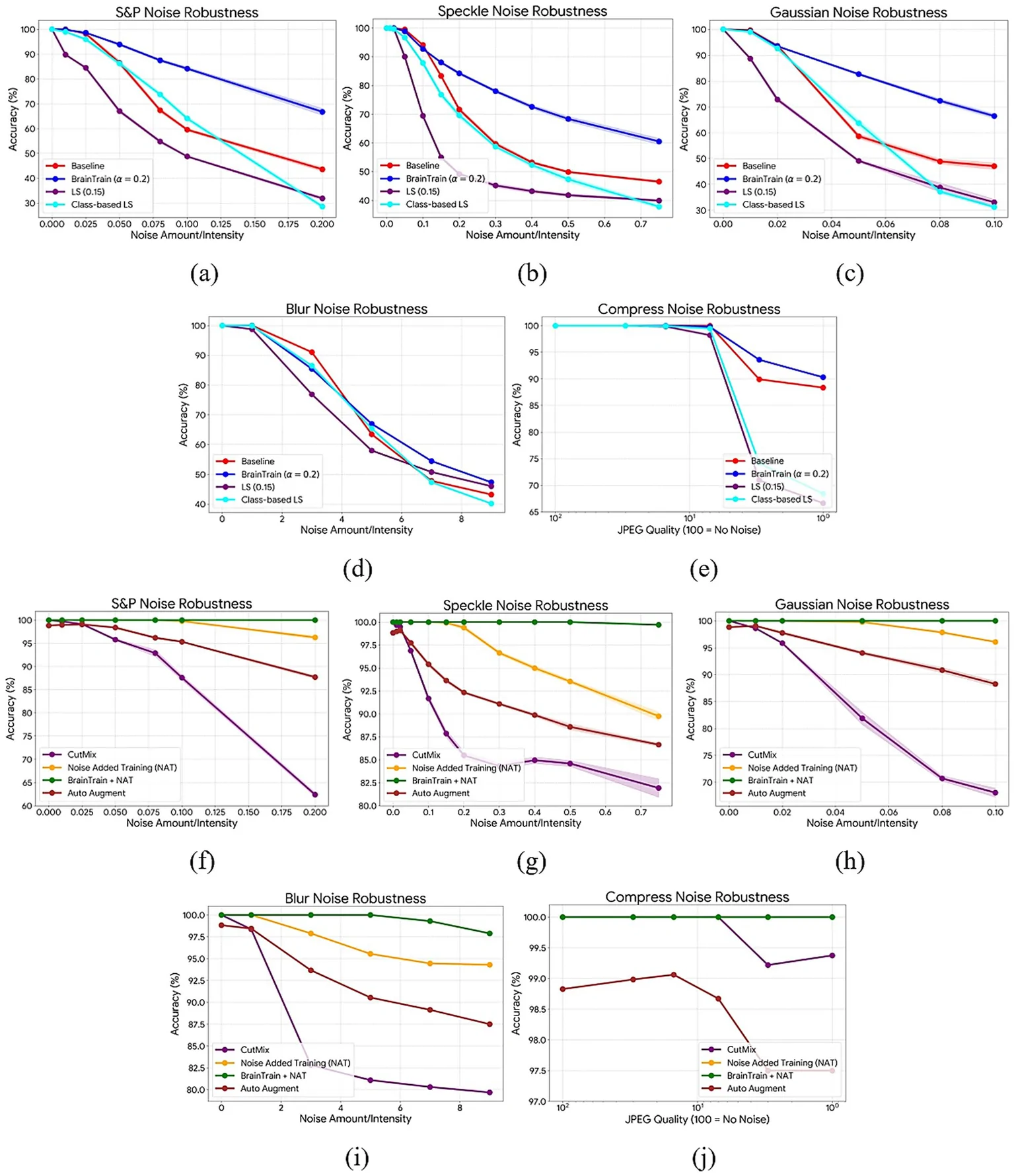
**(a–e)** Accuracy of DNNs trained with standard SGD, label smoothing, class-based label smoothing, and BrainTrain under varying types and levels of input noise. **(f–j)** Accuracy of DNNs produced by noise-added training, CutMix, AutoAugment, and the combination of BrainTrain with noise-added training (BrainTrain+NAT).

Figure 3 and Table 1 present the accuracy of baseline DNNs as well as DNNs trained with BrainTrain under various types and levels of noise. As shown in Figures 3a–e, all models have very high clean accuracy, but under input noise, BrainTrain outperforms the baseline models trained with standard SGD, with label smoothing, and with class-based label smoothing. In fact, uniform label smoothing and class-based label smoothing do not significantly improve upon the baseline, with the former having worse accuracy for noisy inputs in some cases.

**Table 1:** Summary of noise-robustness results for the baselines and BrainTrain.

Methodology	Accuracy under highest noise level for each type (↑)					
	Clean	Gaussian (0.1)	S&P (0.2)	Speckle (0.75)	Gaussian Blur (9)	JPEG Comp. (1)
No augmentation, 6 epochs of finetuning						
Baseline	100	47.06 ± 1.30	43.61 ± 0.88	46.52 ± 0.39	43.20	88.36
Label-smoothing (*ε* = 0.15)	100	33.09 ± 1.20	31.90 ± 0.69	39.94 ± 0.45	46.02	66.64
Class-based LS (*ε* = 0.1, 0.05)	100	31.27 ± 0.58	28.63 ± 0.82	37.83 ± 0.60	40.156	68.44
BrainTrain (Ours)	100	66.44 ± 0.92	66.77 ± 1.37	60.52 ± 1.06	47.34	90.31
With augmentation, 40 epochs of finetuning						
NAT	100	96.09 ± 0.15	96.26 ± 0.36	89.76 ± 0.45	94.30	100
CutMix	100	68.08 ± 0.81	62.38 ± 0.50	81.94 ± 1.00	79.69	99.38
AutoAugment	98.83	88.28 ± 0.49	87.71 ± 0.36	86.66 ± 0.19	87.50	97.50
BrainTrain + NAT (Ours)	100	100.00 ± 0.00	100.00 ± 0.00	99.70 ± 0.05	97.89	100

BrainTrain displays larger accuracy gains over the standard SGD baseline for inputs with S&P (7.41 ± 1.16 to 23.16 ± 1.63 for noise amounts ranging from 0.05 to 0.2, *p* < 0.001), Gaussian (19.38 ± 1.59 to 24.02 ± 1.02 for noise amounts ranging from 0.05 to 0.1, *p* < 0.001), and speckle noise (12.59 ± 0.87 to 19.38 ± 0.90 for noise amounts ranging from 0.2 to 0.75, *p* < 0.001), while performance on other noise types show marginal gains or similar performance.

Figures 3f–j present robustness results for methods that require data augmentation and substantially increase training time. Specifically, we evaluate BrainTrain when combined with noise-added training (BrainTrain+NAT), noise-added training alone (NAT), AutoAugment, and CutMix. Both NAT and BrainTrain+NAT are trained on S&P noise, with the resulting models being evaluated on all noise types. Overall, we see that BrainTrain+NAT clearly performs the best, showing little to no degradation even under heavy noise and unseen noise types (gaussian, speckle, blur, compress). NAT alone performs well but does not match the robustness of the BrainTrain + NAT model and degrades faster on unseen noise types. CutMix displays the largest drop in accuracy under all noise types. AutoAugment displays improvements over CutMix, but does not perform as well as NAT or BrainTrain+NAT. BrainTrain+NAT improves upon NAT’s accuracy for inputs with S&P noise (3.47 ± 0.36 at the highest level of noise, *p* < 0.001), as well as unseen noise types like Gaussian noise (3.91 ± 0.15 at the highest level of noise, *p* < 0.001), speckle noise (9.94 ± 0.45 at the highest level of noise, *p* < 0.001), and Gaussian blur (3.59 at the highest level of noise).

**Figure 4:**
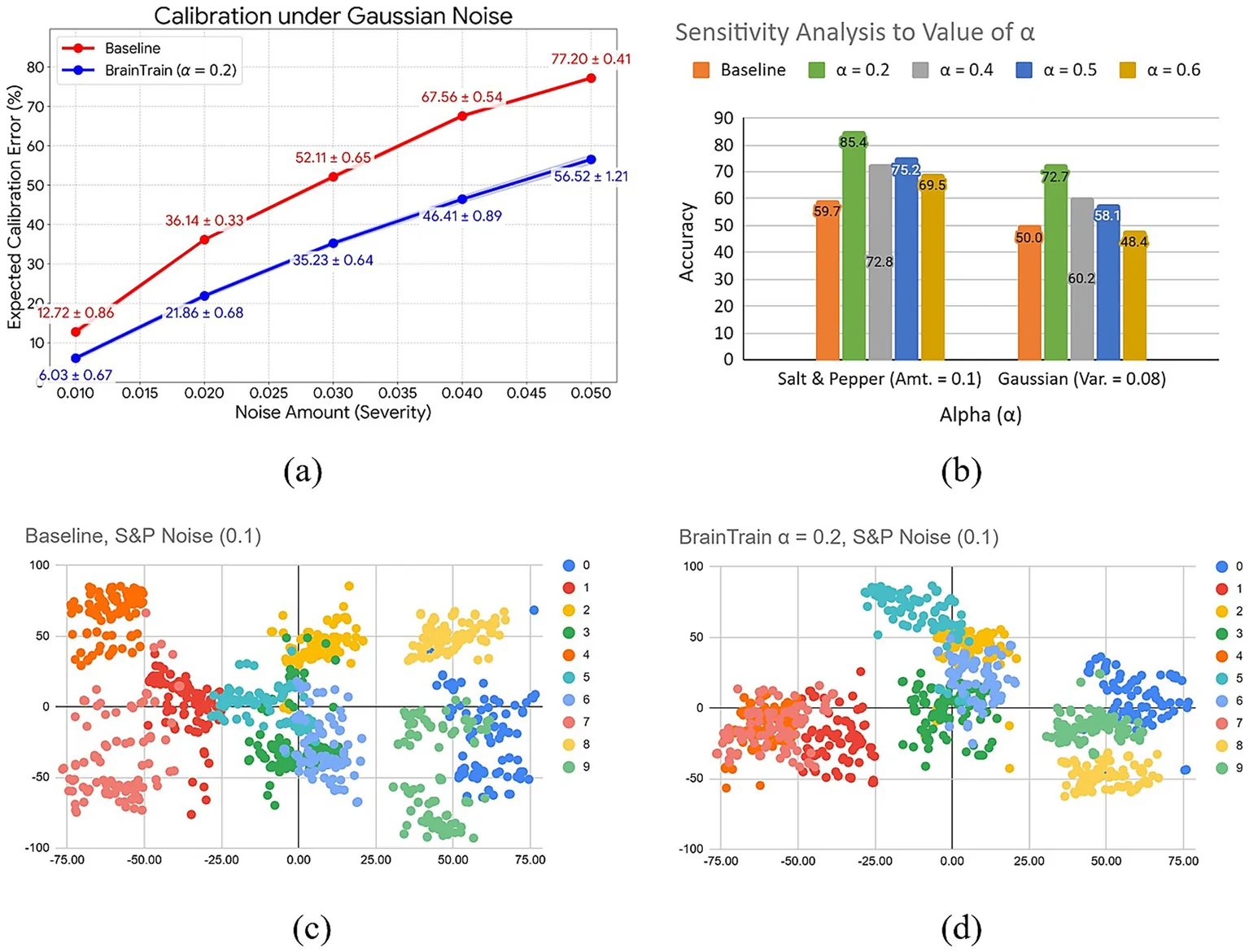
**(a)** Calibration error of DNNs trained with BrainTrain and conventional training across various levels of Gaussian noise; **(b)** sensitivity of the accuracy of BrainTrain to different values of the hyper-parameter *α*. We observe that α = 0.2 provides the best results. Visualization of internal activations of last hidden layer in **(c)** baseline DNNs and **(d)** DNNs trained with BrainTrain using *t*-distributed stochastic neighbor embedded (*t*-SNE).

Figure 4a presents the results of our experiment evaluating calibration error. The x-axis in the figure represents different epochs of training, while the y-axis represents the Expected Calibration Error (ECE). We see that BrainTrain consistently improves calibration error by 1.4× to 2.1× across various levels of Gaussian noise. This improvement arises from a reduction in under-confident correct predictions and over-confident mis-predictions.

Subsequently, we perform a sensitivity analysis to the value of the hyper-parameter *α*, which controls the strength of human behavior alignment used during training. The results in Figure 4b indicate that various values of *α* (0.2, 0.4, 0.5 and 0.6) result in an improvement in accuracy under noisy inputs. However, the *α* = 0.2 case consistently performs the best. Although Figure 4b specifically presents results for two noise types, the trends we observed are consistent across different noise types and levels.

We perform an experiment to help us better understand the improvements in robustness resulting from BrainTrain. DNNs learn high-dimensional internal representations of the input that are successively refined by each hidden layer in the neural network before being projected to the output by the final linear layer(s). We hypothesize that the internal representations of DNNs trained with BrainTrain are less impacted by noise. To validate this hypothesis, we use t-distributed Stochastic Neighbor Embedding (t-SNE) (van der Maaten and Hinton, 2008) to project the activations of the last hidden layer of the ResNet-18 DNN onto two dimensions (for ease of visualization). This is repeated for both the baseline models and BrainTrain with 1,280 input images. The results are presented in Figures 4c,d, with Figure 4c corresponding to the conventional training and Figure 4d corresponding to BrainTrain. In these graphs, the *x* and *y* axes represent the two embedded dimensions onto which the activations of the DNN’s last hidden layer have been projected. Each point represents an input image, and the color represents the actual class of the input. We observe that in the presence of noise, the BrainTrain model better preserves class separation (average point-to-centroid distance of 72.4) compared to the conventionally trained model (average point-to-centroid distance of 79.0), which is consistent with the observed improvement in robustness.

Finally, we evaluate SDLS on the ImageNet dataset, with 1,000 classes, 1.2 million training images, and 50,000 validation images. A vision transformer (ViT-L16) pretrained on ImageNet was used to generate the confusion matrix in place of human responses. The target model (ResNet-50) was trained using a process similar to BrainTrain. When tested on varying levels of Gaussian noise (Figure 2b), SDLS resulted in increased robustness compared to the baseline model trained with conventional SGD. In addition, SDLS combined with noise-added training improved robustness compared to noise-added training alone. In both cases, the increase in accuracy under noisy inputs is around 3%.

## 4 Discussion, limitations, and future work

Our work demonstrates that human behavior alignment has the potential to improve the robustness of DNNs to input noise. While we believe our results are promising, we would like to mention several limitations and future directions that could be pursued. The collection of human behavioral data is time consuming and expensive. While the mobile app ORCA could help, deploying it at scale for data collection is an important direction of future effort. BrainTrain also aggregates human responses at the class-level, not the sample-level. A future direction could involve using sample-wise targets derived from human behavioral data. Our work focuses on object recognition. Extending this approach to other computer vision applications as well as other domains such as natural language processing is an open challenge. Although our results on similarity-driven label smoothing (SDLS) demonstrate some improvements, they are modest compared to the improvements from aligning to human behavioral data. Investigating the cause of this difference and bridging it, as well as evaluating on more comprehensive benchmarks such as ImageNet-C is an important direction that could be explored by future work. While we specifically use human responses to object recognition challenges as our human behavior data, the use of other modalities such as fMRI or EEG recordings could yield new insights and improvements. Our best results are obtained when BrainTrain is combined with noise-added training. Given the large body of work in DNN robustness, combining BrainTrain with other techniques could be similarly fruitful. Finally, our focus in this work is only on naturally occurring noise types, not adversarial perturbations. Humans are also known to be robust to many adversarial inputs, hence future work could also investigate whether a similar approach could be used to improve adversarial robustness.

## Data Availability

The data analyzed in this study is subject to the following licenses/restrictions: Human behavioral data in the form of anonymized human responses to object recognition tasks was obtained from Dr. Michael Lee at MIT (Michael J. Lee, Rapid visual object learning in humans is explainable by low-dimensional image representations, PhD dissertation, Massachusetts Institute of Technology, 2022). The dataset has not been made publicly available. Requests to access these datasets should be directed to Dr. Michael Lee, mil@mit.edu.
